# Identification of potential prognostic TF‐associated lncRNAs for predicting survival in ovarian cancer

**DOI:** 10.1111/jcmm.14084

**Published:** 2018-12-13

**Authors:** Qiuyan Guo, Yanan He, Liyuan Sun, Congcong Kong, Yan Cheng, Peng Wang, Guangmei Zhang

**Affiliations:** ^1^ The First Affiliated Hospital Harbin Medical University Harbin China; ^2^ College of Bioinformatics Science and Technology Harbin Medical University Harbin China

**Keywords:** lncRNAs, ovarian cancer, prognostic signature, survival analysis, transcription factors

## Abstract

The pathophysiology of ovarian cancer (OV) is complex and depends on multiple biological processes and pathways. Therefore, there is an urgent need to identify reliable prognostic biomarkers for predicting clinical outcomes and helping personalize treatment of OV. A long non‐coding RNA (lncRNA)‐based risk score model was constructed to infer the prognostic efficacy of transcription factors (TFs) based on the OV dataset from The Cancer Genome Atlas. The risk score model was further validated in other independent cohorts from Gene Expression Omnibus. Time‐dependent receiver operating characteristic curves were used to evaluate the survival prediction performance in comparison with other clinical and molecular variables. Our results revealed that the top‐ranked TF‐associating lncRNAs were significantly associated with overall survival, progression‐free survival and disease‐free survival. Stratification analysis according to clinical variables indicated the prognostic independence of POLR2A‐associating lncRNAs. In comparison, the signature of POLR2A‐associating lncRNAs was more sensitive and specific than existing clinical and molecular signatures. Functional and experimental analysis suggested that POLR2A‐associating lncRNAs may be involved in known biological processes and pathways of OV. Our findings revealed that the lncRNA‐based risk score model can provide helpful information on OV prognosis stratification and discovery of therapeutic biomarkers.

## 1 INTRODUCTION

Ovarian cancer (OV) is the most common and lethal gynaecological malignant tumour worldwide.^1^ Most OV patients have already developed metastases by the time they are first diagnosed. Despite advances in continuous study and treatment, the prognosis for OV patients remain unsatisfactory, with only 30% of 5 year survival after first line treatment.^2^ Although our understanding of OV is continuously growing, the precise molecular mechanisms underlying this malignant disease are far from understood. The pathophysiology of OV tumour development is complex and depends on multiple biological processes and pathways. Therefore, there is an urgent need to identify reliable prognostic biomarkers for predicting clinical outcomes and personalizing treatment.

Transcription factors (TFs) perform key functions in controlling the expression of coding and non‐coding RNAs by binding to either enhancer or promoter regions. By regulating oncogenes and tumour suppressors, the differential expression of TFs and their downstream targets have been found to associate with the progression of multiple types of cancers.^3^ The expression of long non‐coding RNAs (lncRNAs), which are longer than 200 nucleotides, is also under the control of TFs. Emerging evidence has shown that lncRNAs act as important regulators in diverse physical and pathological tumour processes^4^ and associate with the prognosis of OV patients.^5^

Previous studies have found that TFs are highly selective in regulating targets across different types of tissues and diseases.^6^ Thus, TFs may play different regulatory roles, which can be inferred by their targets in the context of certain tumour microenvironments. Despite the importance of TFs and lncRNAs in cancer development and carcinogenesis, there has only been limited work studying TF‐lncRNA regulation and further evaluating the effects of these interactions on the prognosis of OV. With matched clinical information and genome‐wide expression profiles of coding and non‐coding RNAs, large‐scale lncRNA‐based analysis of TF regulation as possible prognostic biomarkers is now possible.

In the present study, we aimed to identify expression signatures that predict the survival of OV patients. The lncRNA‐based risk score model was constructed to infer the prognostic efficacy of each TF based on the OV dataset from The Cancer Genome Atlas (TCGA).^7^ According to the lncRNA‐based risk score, we found that panels of lncRNAs regulated by the same TFs were significantly associated with patient survival. The risk score model was further validated in the Gene Expression Omnibus (GEO) dataset. Our results indicated that panels of lncRNAs regulated by the same TFs were significantly associated with overall survival (OS), progression‐free survival (PFS) and disease‐free survival (DFS) of patients. Further analysis revealed that the signature of POLR2A‐associating lncRNAs was more sensitive and specific than the existing clinical and other molecular signatures in predicting survival. In summary, our findings revealed that the lncRNA‐based risk score model can provide helpful information on OV prognosis stratification and discovery of therapeutic biomarkers.

## 2 MATERIALS AND METHODS

### 2.1 Clinical and expression OV dataset

The RNA‐sequencing expression profile and related clinical information of 399 serous ovarian carcinoma patients were obtained from the TCGA data portal,^7^ which included 29 250 mRNAs and 10 412 lncRNAs (GENCODE v19). Another three independent OV datasets, GSE26193 (n = 107), GSE9891 (n = 278) and GSE63885 (n = 75) were downloaded from the publicly available Gene Expression Omnibus (GEO) database. Patients with well annotated follow‐up information were retained. For GEO dataset, all profiles were performed based on the same microarray platform (Affymetrix HG‐U133_Plus_2.0 array). To obtain lncRNA expression, probes were remapped to the human genome GENCODE reference (v19) using a previously published pipeline.^8^ SeqMap tool was used to map probes to mRNA and lncRNA sequences.^9^ We performed the “seqmap” command by using default parameters. Probes that were uniquely mapped with no mismatches were retained for further analysis. For probes that mapped to the same gene, the arithmetic mean expression value was used. For mRNAs, a number of 31 811 probes were uniquely mapped. For lncRNAs, a number of 4167 probes were uniquely mapped. Finally, a total of 16 345 mRNAs and 3308 lncRNAs were identified from the microarray data.

### 2.2 Identification of TF‐lncRNA regulatory interactions

TF‐lncRNA interactions were derived from our previous studies,^10^, ^11^ which identified TF binding sites of lncRNAs from 690 ChIP‐Seq datasets across different cell lines and tissues. Furthermore, Pearson's correlation coefficients were calculated for each of the potential TF‐lncRNA pairs based on their expression in TCGA OV patients. We used a Pearson's coefficient >0 and false discovery rate (FDR) <0.05 as the thresholds to identify a link between TFs and lncRNAs. Finally, 4399 potential TF‐lncRNA interactions among 145 TFs and 1234 lncRNAs were identified.

### 2.3 Statistical analysis

Univariate and multivariate Cox regression analyses were performed to evaluate the association between survival and expression of lncRNAs in each OV cohort. In the lncRNA‐based risk score model, the risk score for each patient was calculated according to the linear combination of the expression values weighted by the coefficient from univariate Cox regression analysis:

(1) $$\text{RiskScore}=\sum_{i=1}^{n}\beta_{i}\text{Exp}\left({\text{lnc}}_{i}\right)$$

where β_*i*_ is the Cox regression coefficient of a lncRNA and *n* is the number of lncRNAs regulated by the same TF. Exp(lnc_*i*_) is the expression value of lncRNA *i* in the corresponding patient. The median risk score was used as a cut‐off point to divide the patients into high and low risk groups. Kaplan‐Meier survival curves were plotted for patients in different risk groups, and statistical significance was assessed by the log‐rank test (*P* < 0.05). Patients with censor values were plotted as mark “+.” In survival analysis, the PFS is the length of time during and after the treatment of a cancer that a patient lives with the disease but it does not get worse. The DFS is the length of time after primary treatment for a cancer ends that the patient survives without any signs or symptoms of that cancer. Student's *t* test was used to identify differentially expressed lncRNAs between different groups. All statistical analyses were accomplished based on R framework (v3.4).

### 2.4 Functional analysis

The Enrichr web‐based tool (http://amp.pharm.mssm.edu/Enrichr/) was used to perform functional annotation of lncRNAs.^12^, ^13^ Gene Ontology (GO) terms and Kyoto Encyclopaedia of Genes and Genomes (KEGG) pathways with FDR <0.05 were considered to be significantly enriched. The Cytoscape plugin software, EnrichmentMap, was used for visualization and interpretation of functional annotations.^14^

### 2.5 Ovarian cancer cell line and tissues

The SKOV3 cell line of OV was maintained in Roswell Park Memorial Institue‐1640 medium supplemented with 10% fetal bovine serum (FBS; Ausbian, Austria) at 37°C in 5% CO_2_ and routinely passage at 2‐ to 3‐day intervals. A number of eight OV tissues were collected from eight patients with 5‐year follow‐up information under surgery at the First Affiliated Hospital of Harbin Medical University after written informed consent was obtained from each patient. In addition, three pairs of OV and normal ovary tissues were also collected. The study was approved by the Ethics Committee of the First Affiliate Hospital of Harbin Medical University. Tissues from all patients were without chemotherapy before operation. Both OV and normal tissues were immediately frozen in liquid nitrogen for subsequent experiments.

### 2.6 RNA isolation, reverse transcription PCR, and real‐time PCR

Total RNA was extracted from normal/tumour tissues and cell line using the TRIzol reagent (Invitrogen, USA). The total RNA was used only if the A260/280 ratio of the absorbances ranged between 1.8 and 2.2 as determined by spectrophotometry. Real‐time quantification lncRNA was performed in a 20 mL SYBR reaction system with the SYBR premix ExTaqTM II kit (TAKARA, Japan), and the cycle threshold (Ct) of each gene was recorded. All quantifications were performed with GAPDH as the internal standard and calculated using the 2^−ΔΔCt^ method (ΔCt = Ct^target gene^ − Ct^internal control^). The real‐time polymerase chain reaction (PCR) conditions were as follows: 95°C 10 minutes; 40 cycles of 95°C 30 seconds, 60°C 1 minute, and 95°C 1 minute, 55°C 30 seconds, 95°C 30 seconds. Each sample was measured in triplicate. Primer sequences used in our study are listed in Table S1.

### 2.7 Cell proliferation assay

Cell proliferation was assessed by the Cell Counting Kit‐8 (CCK‐8; Dojindo, Japan). Cells were seeded in 96‐well plates (4000 cells per well) after 48 hours post‐transfection with 100 μL full culture medium. The cells were transfected with si‐NC, si‐KIF25‐AS1, si‐LINC01355 and si‐AC092171.2 for 48 hours. 10 μL of CCK‐8 solution was added to each well, and the plates were incubated for 4 hours in 37°C. Absorbance was read at a wavelength of 450 nm by a microplate reader (ELX800; Bio‐Tek, Ameria). Three independent experiments were performed triplicate.

### 2.8 Wound healing assay

Cells transfected with si‐NC, si‐KIF25‐AS1, si‐LINC01355 and si‐AC092171.2 were seeded into six‐well culture plates with serum‐containing medium and were cultured until the cell density reached 90%‐95% confluence. An artificial homogeneous wound was created by scratching the monolayer with a sterile 200 μL pipette tip. After scratching, the cells were washed with PBS, and then the cells were cultured with serum‐free RPMI‐1640 media for 48 hours. Images of cells migrating into the wound were captured at 0 and 48 hours using a microscope (EVOS, USA). The assay was performed in triplicate.

### 2.9 Transient transfection

The si‐KIF25‐AS1, si‐LINC01355 and si‐AC092171.2 and negative control siRNA (NC) were obtained from RiboBio (Guangzhou, China). The SKOV3 cells were plated onto a six‐well plate and allowed to adhere overnight. Then, the OV cells were transfected with each siRNA using the riboFectTM CP transfection kit (RiboBio) according to manufacturer's instructions for 48 hours used for subsequent experiment.

## 3 RESULTS

### 3.1 Construction of the lncRNA‐based risk score model

**Figure 1:**
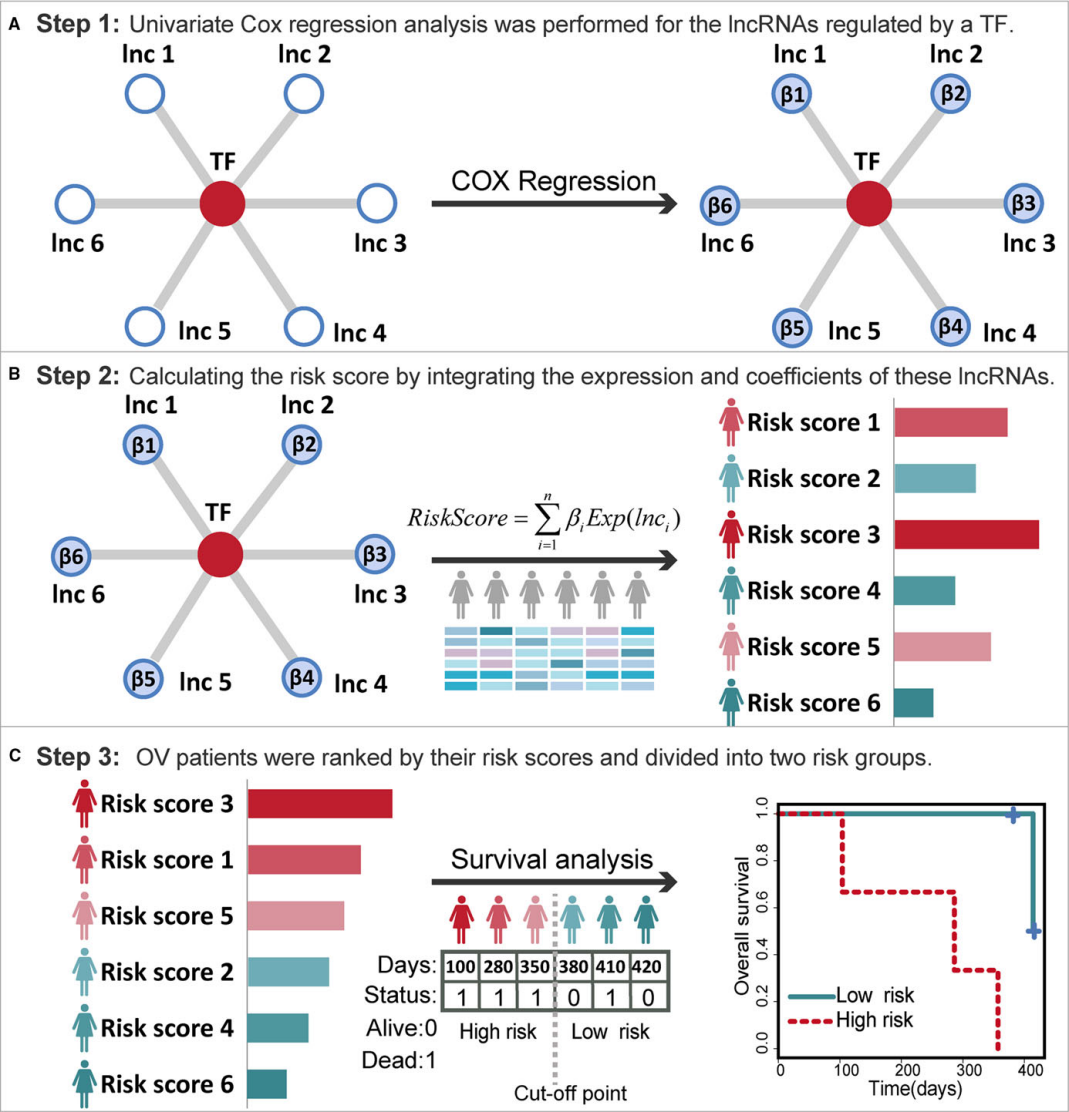
Workflow of the lncRNA‐based risk score model for evaluating the prognostic ability of TFs

A total of 399 OV samples from the TCGA dataset were used for model construction. The TF‐lncRNA regulations were derived from our previous studies,^10^, ^11^ in which we developed an integrated pipeline to predict functional TF‐lncRNA regulatory interactions. In co‐expression analysis, the Pearson's coefficient >0 and FDR <0.05 were used as thresholds to identify links between TFs and lncRNAs. Consequently, we obtained 4399 potential TF‐lncRNA interactions among 145 TFs and 1234 lncRNAs. The major concept of our lncRNA‐based risk score model is to evaluate the prognostic efficiency of a set of lncRNAs, which were regulated by the same TF. There were three general steps in the workflow of the lncRNA‐based risk score model (Figure 1A‐C). In step 1, univariate Cox regression analysis was performed for the lncRNAs regulated by a TF (Figure 1A). In step 2, a risk score formula was developed by integrating the expression values and corresponding coefficients of these lncRNAs (Figure 1B). Based on this formula, each patient was given a risk score. In step 3, the 399 OV patients were ranked by their risk scores and divided into two risk groups by the median risk score. Further survival analysis was performed to evaluate the prognostic significance between two risk groups (Figure 1C).

### 3.2 Application of the lncRNA‐based risk score model

**Figure 2:**
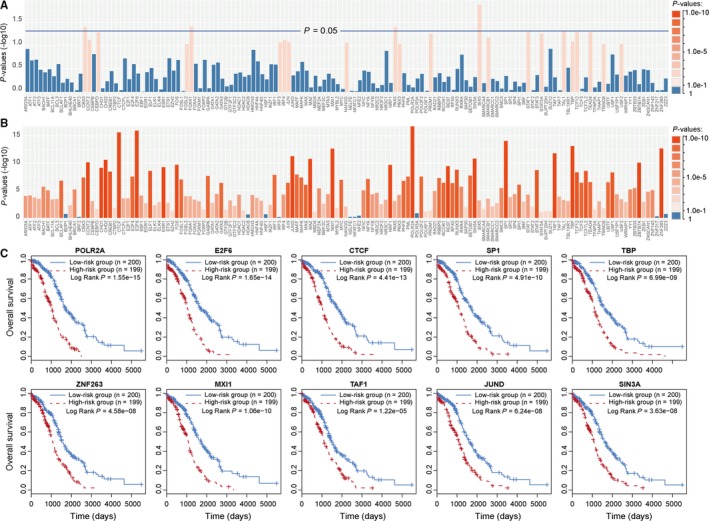
Application of the lncRNA‐based risk score model on TFs. (A) Bar plot of prognostic *P*‐values for TFs before application of the lncRNA‐based risk score model. (B) Bar plot of prognostic *P*‐values for TFs after application of the lncRNA‐based risk score model. *P*‐values were transformed as ‐log10. (C) Kaplan‐Meier survival analysis for each of the top 10 ranked TFs based on the TCGA dataset

Before application of the lncRNA‐based risk score model, we performed univariate Cox regression analysis on each of the 145 TFs. We found that only five TFs (CBX3, FOXA1, PAX5, SIX5 and TAL1) were significantly associated with the prognosis of patients (Figure 2A). Some TFs, such as STAT3 and CTCF, which have been reported as OV prognostic factors,^15^, ^16^ were not significantly associated with prognosis. Then, we applied the lncRNA‐based risk score model to these 145 TFs. Through the model, each TF was given a risk score by evaluating the prognostic efficiency of its regulating lncRNAs. Based on the risk score, we found that most TFs were significantly associated with patient prognosis after application of the lncRNA‐based risk score model (Figure 2B). Some known OV‐associated TFs, such as CTCF and POLR2A, were ranked among the top 10 TFs based on *P*‐values (Table S2). POLR2A, which was the top‐ranked TF, was found to be significantly associated with prognosis (HR = 1.68, 95% CI = 1.50‐1.87). Furthermore, we performed survival analysis for each of the top 10 TFs based on the risk scores resulting from the risk model. Then, 399 TCGA OV patients were assigned into a high risk group (n = 199) or a low risk group (n = 200) by using the median risk score as the cut‐off point. The result of Kaplan‐Meier analysis showed significant differences in patient OS between high and low risk groups (log‐rank test *P* < 1.0E‐5, Figure 2C). These results indicated that our lncRNA‐based model effectively evaluated the prognostic efficacy of TFs.

### 3.3 Validation of the lncRNA‐based risk model in other independent cohorts

**Figure 3:**
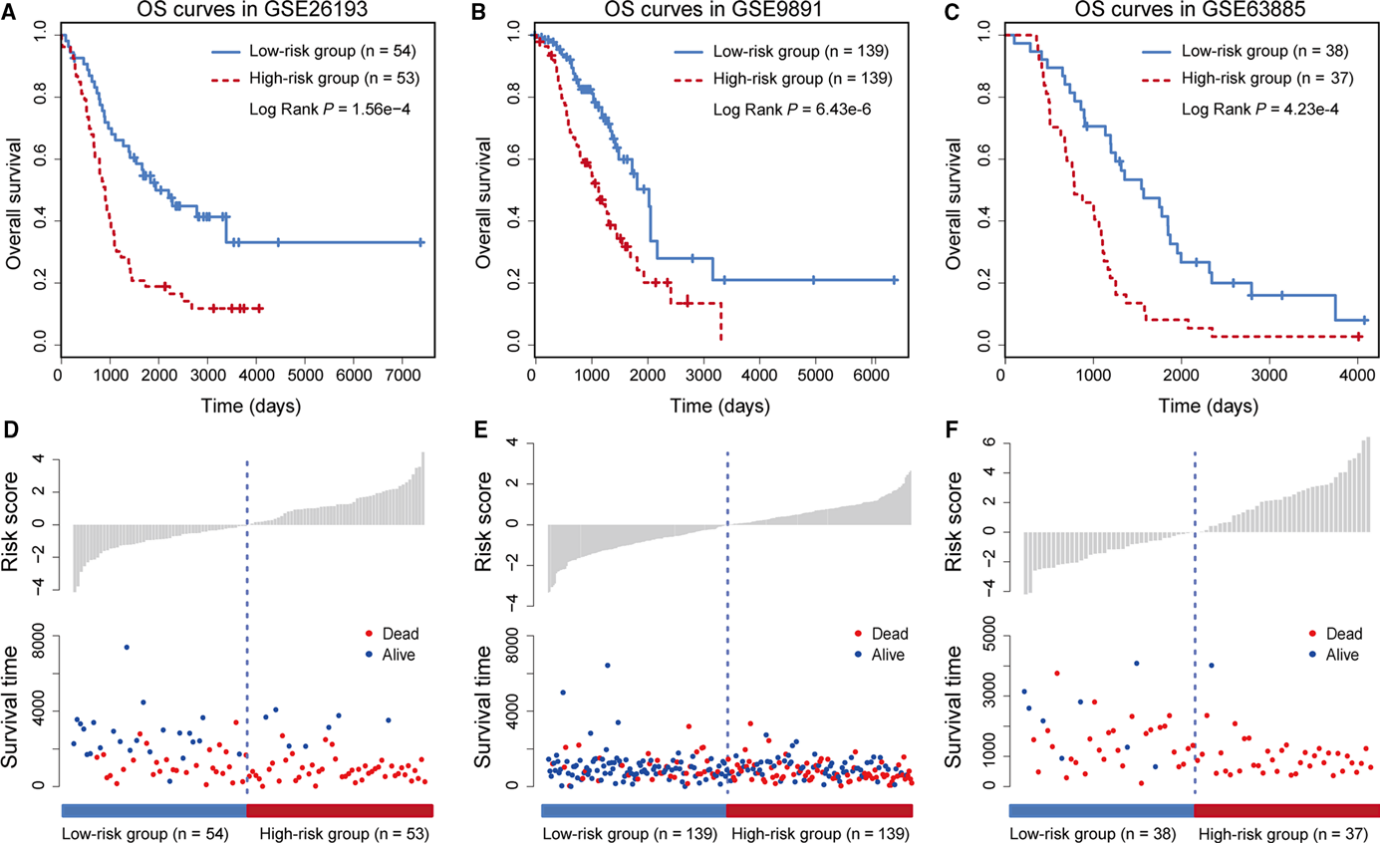
OS analysis for POLR2A‐lncRNAs based on other independent datasets. (A) The OS curves for GSE26193. (B) The OS curves for GSE9891. (C) The OS curves for GSE63885. (D‐F) The distribution of risk scores (normalized by minus median value) and survival time in low and high risk groups of patients

To further evaluate the prognostic efficacy of the lncRNA‐based risk score model, we applied it on another three independent OV datasets: GSE26193 (n = 107), GSE9891 (n = 278) and GSE63885 (n = 75). LncRNAs regulated by the same TFs were used to calculate the risk score. Through the same model construction as for the TCGA dataset, patients in these independent datasets were given risk scores and classified into high or low risk groups. LncRNAs regulated by POLR2A, which was the top‐ranked TF in the TCGA dataset, were found to be significantly associated with prognosis in GSE26193 (HR = 1.48, 95% CI = 1.26‐1.72, *P* = 1.08E‐6), GSE9891 (HR = 1.77, 95% CI = 1.46‐2.15, *P* = 5.73E‐9) and GSE63885 (HR = 1.29, 95% CI = 1.16‐1.43, *P* = 4.02E‐6). Based on the median risk score of POLR2A target lncRNAs, patients in the three GEO datasets were divided into high and low risk groups, respectively. Significant association between the risk score and OS was observed in all three independent datasets (Figure 3A‐C). The distribution of patient risk scores and survival status is shown in Figure 3D‐F. Furthermore, we used the same cut‐off point identified from TCGA dataset to divide GEO datasets for the purpose of validation. All of the three independent datasets could be significantly divided into high and low risk groups (Figure S1A‐C). In this step, we used a *z*‐score normalization method to normalize the risk scores in different dataset. Patients with higher risk scores tended to have shorter survival time, whereas patients with lower risk scores tended to have longer survival time. Cox regression hazards and Kaplan‐Meier survival curves for lncRNAs regulated by the top 10 TFs were shown in Table S2 and Figure S2. We found that most TFs could significantly divided patients into different risk groups. These observations were consistent with the findings in the TCGA dataset.

### 3.4 Prognostic performance of top‐ranked TFs on PFS/DFS

**Figure 4:**
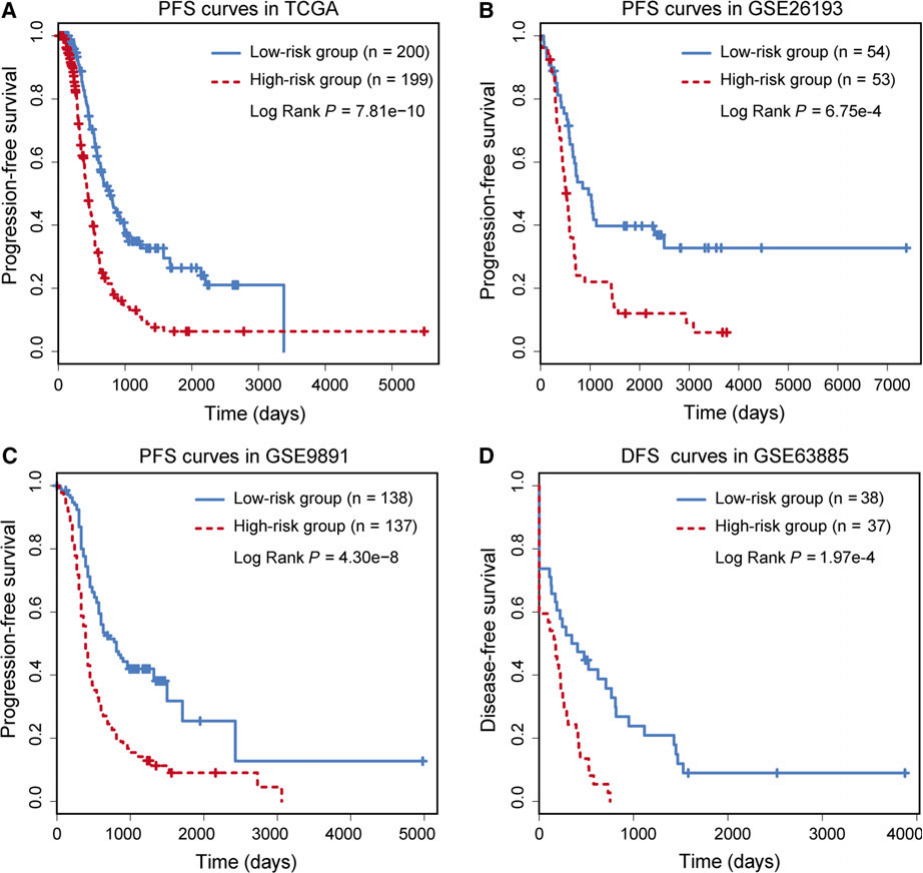
PFS or DFS analysis for POLR2A‐lncRNAs based on the TCGA and GEO datasets. (A) The PFS for TCGA. (B) The PFS for GSE26193. (C) The PFS for GSE9891. (D) The DFS for GSE63885

Moreover, we evaluated the prognostic performance of the top‐ranked TFs on PFS and DFS. PFS analysis was performed on the TCGA dataset (n = 399), GSE26193 (n = 107) and GSE9891 (n = 275), while the DFS analysis was performed on GSE63885 (n = 75). In this step, three patients with missing follow‐up values in GSE9891 dataset were excluded. The POLR2A‐associated lncRNAs (POLR2A‐lncRNA) signature was found to be significantly associated with patient PFS in the dataset of TCGA (HR = 1.63, 95% CI = 1.45‐1.84, *P* = 1.11E‐15), GSE26193 (HR = 1.54, 95% CI = 1.31‐1.81, *P* = 1.29E‐7) and GSE9891 (HR = 1.84, 95% CI = 1.54‐2.20, *P* = 1.56E‐11). In GSE63885, the POLR2A‐lncRNA signature was significantly associated with patient DFS (HR = 1.48, 95% CI = 1.24‐1.77, *P* = 1.59 E‐5). Significant association between risk scores and PFS/DFS was observed in all four datasets (Figure 4A‐D). Furthermore, we used the cut‐off point identified from TCGA dataset to divide GEO datasets for PFS and DFS analysis. All of the three independent datasets could also be significantly divided into high and low risk groups (Figure S3A‐C). The POLR2A‐lncRNA signature successfully divided the OV patients into high and low risk groups. For each of the top 10 TFs, Cox regression hazards and Kaplan‐Meier survival curves of PFS/DFS are shown in Table S3 and Figures S4‐S7. All 10 TFs were significantly associated with patient PFS/DFS (*P* < 0.01).

### 3.5 Independence of POLR2A‐lncRNA signature from clinical variables

**Figure 5:**
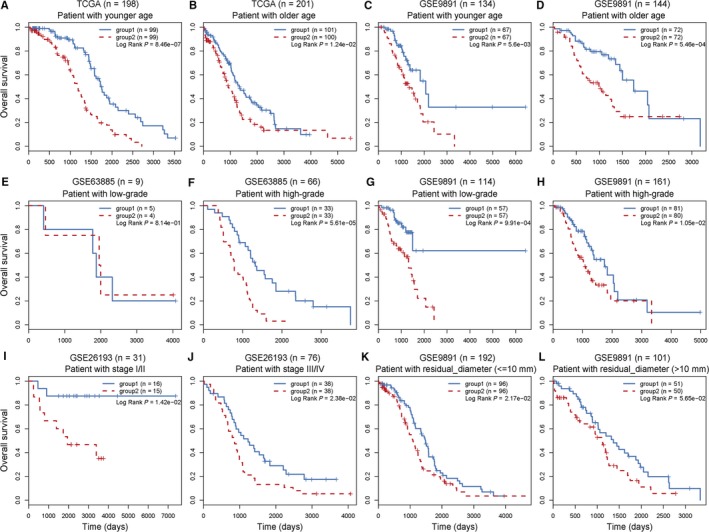
Survival analysis of patients with available clinical information. (A, B) Kaplan‐Meier curves for patients with younger (A) and older (B) age in TCGA. (C, D) Kaplan‐Meier curves for patients with younger (C) and older (D) age in GSE9891. (E, F) Kaplan‐Meier curves for patients with lower (E) and higher (F) grade in GSE63885. (G, H) Kaplan‐Meier curves for patients with lower (G) and higher (H) grade in GSE9891. (I, J) Kaplan‐Meier curves for patients with stage I/II (I) and III/IV (J). (K, L) Kaplan‐Meier curves for patients with residual tumour diameter ≤10 mm (K) and >10 mm (L)

To test whether the POLR2A‐lncRNA signature was independent of other clinical variables, multivariate Cox regression analyses were performed in each OV cohort. The POLR2A‐lncRNA signature and other clinical and pathological variables such as patient age, tumour stage, tumour grade, residual tumour diameter and lymph node metastasis were analysed (Table 1). The POLR2A‐lncRNAs signature was significantly correlated with survival of patients in TCGA (*P* = 1.26E‐7), GSE26193 (*P* = 6.22E‐5), GSE9891 (*P* = 7.46E‐8) and GSE63885 (*P* = 6.40E‐5). In addition, we found that two clinical variables, patient age and tumour grade, were also significantly associated with survival in at least two OV cohorts. Thus, further stratification analysis according to patient age and tumour grade were performed. The patients in the TCGA and GSE9891 datasets were stratified into younger and older groups according to the median value. We found that the POLR2A‐lncRNA signature significantly subdivided the patients at different age levels into different risk subgroups in the TCGA dataset (Figure 5A,B) and the GSE9891 dataset (Figure 5C,D). For tumour grade, patients in the GSE63885 and GSE9891 datasets were stratified into a low grade (G1/G2) and a high grade (G3/G4) group. In the GSE63885 dataset, the POLR2A‐associating lncRNAs signature significantly subdivided the high grade patients into different risk subgroups (Figure 5E,F). In GSE9891, both low grade patients (log rank *P* = 9.91E‐4) and high grade patients (log rank *P* = 1.05E‐2) were subdivided into different risk subgroups by the signature (Figure 5G,H). Similar results were also observed in stratification analysis of stage and residual tumour diameter variables (Figure 5I‐L).

**Table 1:** Univariate and multivariate Cox regression analysis of the POLR2A‐lncRNA signature and other clinical variables

Datasets	Variables	Univariate analysis		Multivariate analysis	
		HR (95% CI)	*P* values	HR (95% CI)	*P* values
TCGA	Age	1.016 (1.004‐1.028)	1.05E‐02	1.017 (1.004‐1.030)	8.63E‐03
	Stage (I/II/III/IV)	1.309 (0.982‐1.745)	6.66E‐02	1.348 (0.979‐1.855)	6.71E‐02
	Grade (G1/G2/G3/G4/GX)	1.056 (0.805‐1.385)	6.93E‐01	0.976 (0.751‐1.267)	8.53E‐01
	Residual diameter	1.398 (1.164‐1.678)	3.29E‐04	1.295 (1.068‐1.571)	8.59E‐03
	Lymph node metastasis	1.048 (0.868‐1.266)	6.23E‐01	0.903 (0.739‐1.104)	3.21E‐01
	POLR2A‐lncRNAs	1.675 (1.501‐1.870))	0	1.965 (1.530‐2.525)	1.26E‐07
GSE9891	Age	2.152 (1.49‐3.109)	4.44E‐05	2.195 (1.444‐3.336)	2.32E‐04
	Stage (I/II/III/IV)	0.961 (0.895‐1.031)	2.69E‐01	1.092 (0.783‐1.522)	6.04E‐01
	Grade (G1/G2/G3)	1.025 (1.005‐1.046)	1.22E‐02	1.022 (1.001‐1.043)	3.68E‐02
	POLR2A‐lncRNAs	1.774 (1.463‐2.152)	5.73E‐09	1.724 (1.413‐2.102)	7.46E‐08
GSE26193	Stage (I/II/III/IV)	1.207 (0.829‐1.759)	3.26E‐01	2.004 (1.44‐2.789)	3.79E‐05
	Grade (G1/G2/G3)	2.057 (1.546‐2.738)	7.54E‐07	0.683 (0.458‐1.016)	6.00E‐02
	POLR2A‐lncRNAs	1.475 (1.262‐1.724)	1.08E‐06	1.375 (1.177‐1.608)	6.22E‐05
GSE63885	Stage (II/III/IV)	2.315 (1.239‐4.323)	8.45E‐03	1.57 (0.803‐3.069)	1.87E‐01
	Grade (G2/G3/G4)	1.707 (1.129‐2.582)	1.13E‐02	1.558 (1.028‐2.362)	3.68E‐02
	POLR2A‐lncRNAs	1.287 (1.156‐1.432)	4.02E‐06	1.257 (1.124‐1.406)	6.40E‐05

### 3.6 Comparison with established clinical and molecular variables

**Figure 6:**
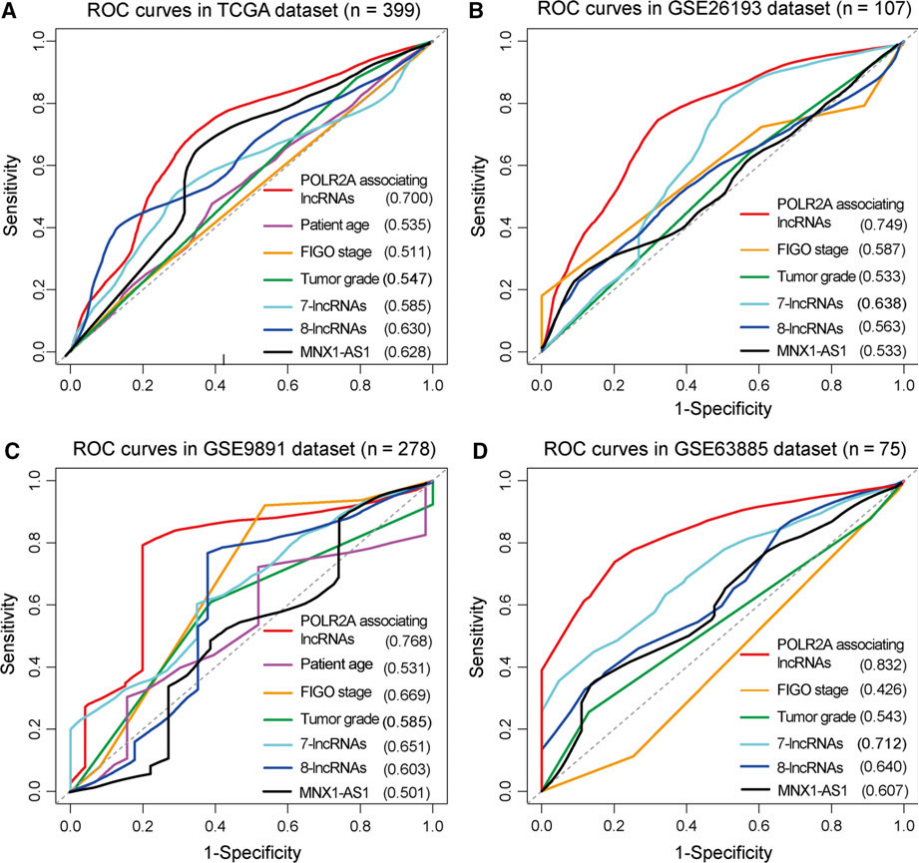
Time‐dependent ROC analysis of the sensitivity and specificity for survival prediction based on the POLR2A‐lncRNAs signature and other variables. (A) ROC curves in TCGA. (B) ROC curves in GSE26193. (C) ROC curves in GSE9891. (D) ROC curves in GSE63885

To assess the sensitivity and specificity of OS prediction between our lncRNA‐based risk score model and other clinical and molecular variables, we performed Time‐dependent receiver operating characteristic (ROC) curve analysis. ROC curves of the POLR2A‐lncRNA signature and other clinical variables including patient age, FIGO stage and tumour grade were compared. ROC curves were also compared with other established risk models including a panel of seven lncRNAs signature (BC037530, AK021924, AK094536, BC062365, AK130460, BC007937 and BC004123),^17^ a panel of eight lncRNAs signature (RP4‐799P18, PTPRD‐AS1, RP11‐57P19, RP11‐307C12, RP11‐254I22, RP11‐80H5, RP1‐223E5 and GACAT3)^18^ and a single lncRNA MNX1‐AS1.^19^ The median survival time was used as a cut‐off threshold to identify positive and negative cases. By comparing the area under the curve (AUC) of ROC, we found that the predictive value of POLR2A‐lncRNA signature was higher than other clinical and molecular variables (Figure 6A‐D). In GSE63885, the POLR2A‐lncRNA signature reached the highest AUC value of 0.832. These observations indicate that our model was more sensitive and specific than existing clinical and molecular signatures in predicting the survival of OV patients.

### 3.7 Functional prediction of POLR2A‐lncRNA

**Figure 7:**
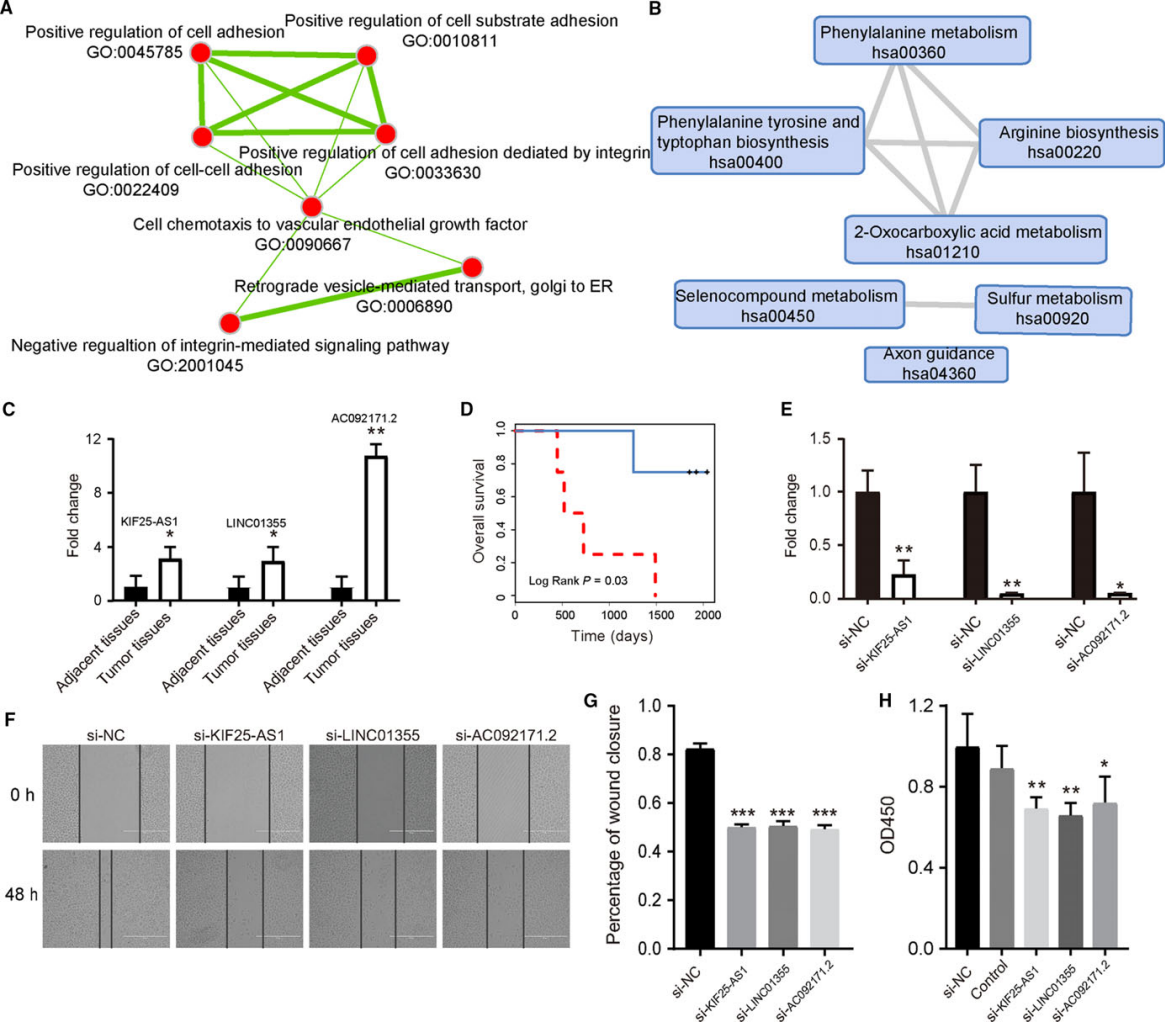
Functional detection of POLR2A‐lncRNAs. (A) Functional map of enriched biological processes. (B) Functional map of enriched KEGG pathways. In the functional maps, each node indicates an enriched GO term or KEGG pathway, and each edge indicates common genes shared between two nodes. (C) LncRNAs KIF25‐AS1, LINC01355 and AC092171.2 were differentially expressed between adjacent and tumour tissues. (D) Through the lncRNA‐based model, integration of KIF25‐AS1, LINC01355 and AC092171.2 could significantly divided eight clinical patients into different risk groups. (E) After transfection with siRNAs, the relative expression of KIF25‐AS1, LINC01355 and AC092171.2 were significantly down‐regulated. (F) Representative images from the results of wound healing assay with siRNAs for KIF25‐AS1, LINC01355 and AC092171.2.(G) Knock down of risk lncRNAs KIF25‐AS1, LINC01355 and AC092171.2 caused a significantly decrease capacity of cell migration. (H) CCK‐8 assays were performed to analyse cell growth after knock down of KIF25‐AS1, LINC01355 and AC092171.2. *P < 0.05; **P < 0.01; ***P < 0.001

Furthermore, we explored the functions of POLR2A‐lncRNA by using the Enrichr tool,^12^, ^13^ which performs a comprehensive gene set enrichment analysis based on different functional contexts such as GO and KEGG pathways (Table S4). We found that a series of GO terms associating with positive regulation of cell adhesion processes were significantly enriched (Figure 7A). Cell adhesion processes have been found to be involved in metastasis progression and are associated with clinical outcomes of OV patients.^20^, ^21^ In pathway analyses, phenylalanine metabolism and other pathways were enriched (Figure 7B). Previous studies have found significant changes in phenylalanine catabolism in metastatic OV tumours.^22^

### 3.8 Experimental validation of prognostic lncRNAs

Among the lncRNAs regulated by POLR2A, nine lncRNAs have the same risk direction (positive or negative of regression coefficients) across four OV datasets (Figure S8). We randomly selected three lncRNAs (KIF25‐AS1, LINC01355 and AC092171.2) to perform experimental validation. We first measured the expression levels of these three lncRNAs using qRT‐PCR in normal and tumour tissues (Section 2). We found that these three lncRNAs were differentially expressed between normal and OV tissues (Figure 7C, Student's *t* test, *P* < 0.05). Further, we tested the prognostic significance of these three lncRNAs by integrating the expression value in eight OV patients with 5‐year follow‐up information. We found that these three lncRNAs could significantly divided patients into different risk groups (Figure 7D, Log‐rank test, *P* < 0.05), which were consistent with the findings in the TCGA and GEO dataset. Then we performed in vitro analysis to determine the roles of these prognostic lncRNAs in OV. Considering these three lncRNAs were risk factors (having positive Cox coefficients across four OV datasets), siRNAs were used to knockdown the expression of these lncRNAs. The SKOV‐3 cells were transfected with si‐NC, si‐KIF25‐AS1, si‐LINC01355 and si‐AC092171.2, respectively. Real‐time PCRs were performed to verify the transfection ratio (Figure 7E). We observed the effects of each lncRNAs on cell migration of SKOV‐3 cells by wound healing assay. The wound healing scratches were observed by phase‐contrast microscope at 0 and 48 hours, which showed that down‐regulated of these risk lncRNAs caused a significantly decrease capacity of cell migration (Figure 7F,G). Then, CCK‐8 assays were performed to investigate the effects of these lncRNAs on cellular proliferation in SKOV‐3 cells (Figure 7H). The results indicated that knock down of these lncRNAs could significantly decrease the proliferation rate of OV cells.

## 4 DISCUSSION

To identify expression signatures that predict the survival of OV patients, we built the lncRNA‐based risk score model to infer the prognostic efficacy of each TF. The prognostic analysis was performed based on the OV dataset from TCGA.^7^ According to the lncRNA‐based risk score, we found that the top‐ranked TFs were significantly associated with survival. The risk score model was further validated in the independent cohorts GSE26193, GSE9891 and GSE63885. We found that most of the TFs significantly divided patients into different risk groups, which were consistent with the findings in the TCGA dataset. Furthermore, the significant association between risk scores and PFS/DFS was observed in all four datasets (Figure 4A‐D). For each of the top 10ranked TFs, Cox regression hazards analysis was performed, and Kaplan‐Meier survival curves of PFS/DFS were constructed. The POLR2A‐lncRNA significantly divided the OV patients into high and low risk groups in terms of OS, PFS and DFS. Initially, a total number of 53 lncRNAs were identified to be regulated by the TF POLR2A (Table S5). Considering that the biological experiments are expensive and time consuming, we performed a bioinformatics analysis by calculating the Cox regression coefficients for each lncRNA in different OV datasets. LncRNAs which had the same risk direction (positive or negative of regression coefficients) across different OV datasets were retained for further analysis. There were nine lncRNAs passed the threshold (Figure S8). Among the nine lncRNAs, three lncRNAs were randomly selected and used for experimental validation.

Univariate and multivariate Cox regression analyses were performed to test whether the top‐ ranked POLR2A‐lncRNA signature was independent of other clinical variables. The HR values revealed that the POLR2A‐lncRNA signature was a negative factor for prognosis of OV (Table 1). We found that two clinical variables, patient age and tumour grade, were also significantly associated with survival in at least two OV cohorts. Stage was significantly associated with survival in GSE26193, and residual tumour diameter was significantly associated with survival in GSE9891. Thus, further stratification analysis according to patient age, tumour grade, stage and residual tumour diameter were performed. We found that the POLR2A‐lncRNA signature could significantly subdivide patients into different risk subgroups within different levels of age, grade, stage and residual tumour diameter. Time‐dependent ROC analysis was performed to assess the sensitivity and specificity of OS prediction between our lncRNA‐based risk score model and other variables. These observations indicated that our model was more sensitive and specific than the existing clinical and molecular signatures in predicting the survival of OV patients.

Considering that re‐annotation of lncRNAs from the microarray dataset cannot cover all lncRNA transcripts, there is a limitation of this study in identification of lncRNAs. In this study, only 3308 lncRNAs were identified from the microarray dataset. Thus, additional potential signatures associating with OV survival may be overlooked. In future studies, increasing numbers of non‐coding signatures could be assessed with survival analysis as the acquisition of matched clinical information and whole genome expression profiles become available. Additionally, further experimental analysis will be needed to validate the exact molecular mechanisms of these potential biomarkers in OV.

In summary, in this study we constructed the lncRNA‐based risk score model to infer the prognostic efficacy of TFs in both RNA‐sequencing and microarray datasets. We found that the top‐ranked TF‐lncRNAs were significantly associated with OV prognosis in OS, PFS and DFS analyses. Further analysis indicated that the POLR2A‐lncRNA signature was independent of other clinical variables and more sensitive and specific than existing clinical and molecular signatures. Our systematic analysis revealed that the lncRNA‐based risk score model can provide helpful information in the discovery of prognostic biomarkers of OV.

## CONFLICT OF INTEREST

The authors confirm that there are no conflicts of interest.

## AUTHOR CONTRIBUTION

GMZ and PW conceived, designed and supervised the overall study; PW, YNH, QYG, LYS, CCK and YC carried out data processing and experimental analysis; GMZ, PW, YNH and QYG drafted the manuscript. All authors read and approved the final manuscript.

## ETHICS STATEMENT

The authors confirm that informed consent was obtained from all subjects, and that the research was carried out according to the World Medical Association Declaration of Helsinki.
