# Supplementary material for: Identification of potential prognostic TF‐associated lncRNAs for predicting survival in ovarian cancer
Source: J Cell Mol Med. 2018 Dec 13;23(3):1840–51. doi: 10.1111/jcmm.14084 (PMC6378234; doi:10.1111/jcmm.14084)
Supplement: Supplementary file 1 [file JCMM-23-1840-s001.pdf]

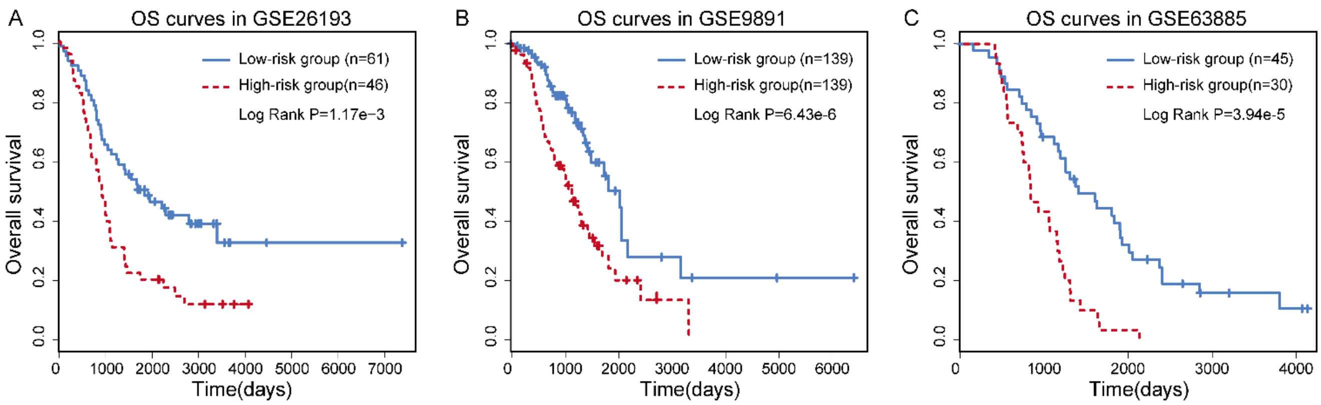

Figure S1. Using the same cut-off point identified from TCGA dataset to divide GEO datasets. (A) The OS curves for GSE26193. (B) The OS curves for GSE9891. (C) The OS curves for GSE63885.

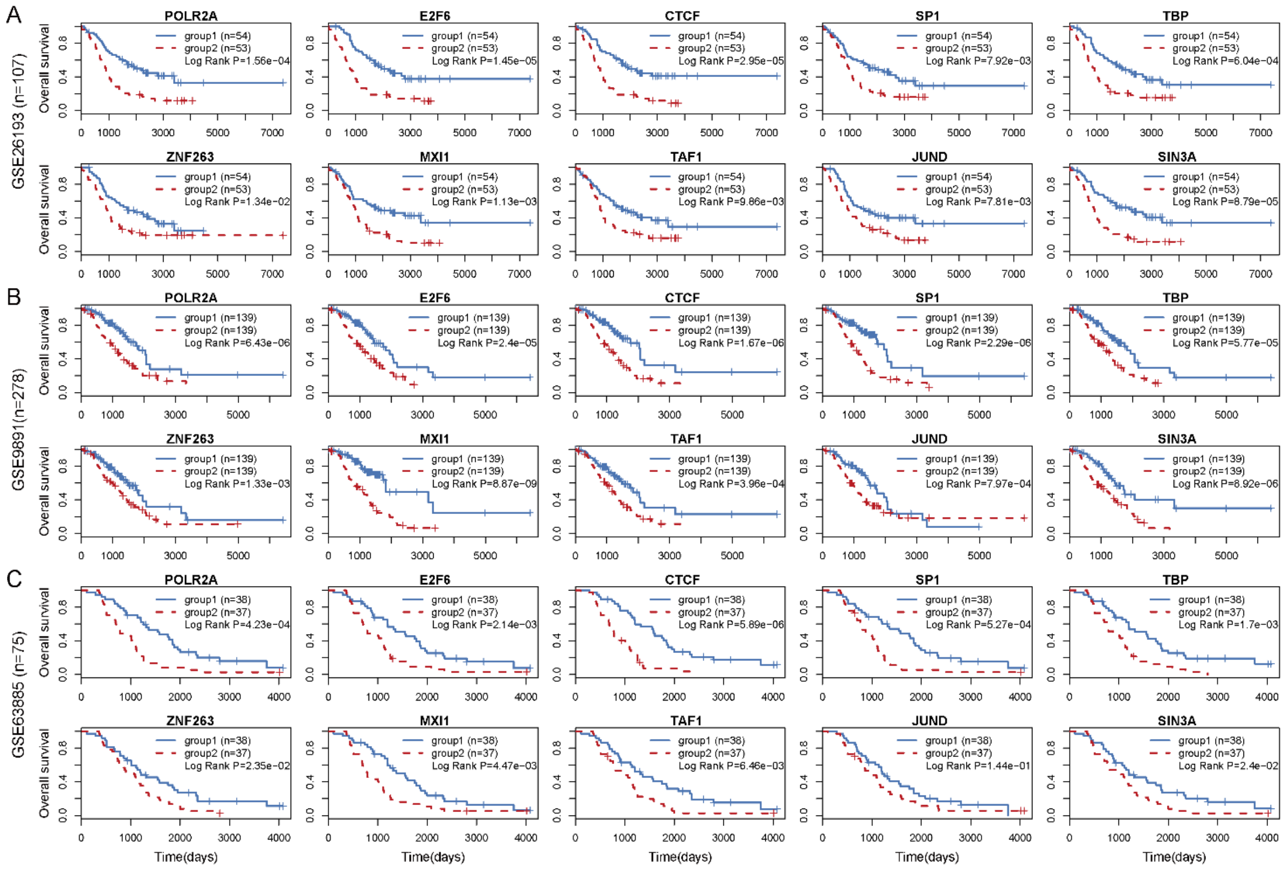

Figure S2. Kaplan-Meier survival curves for lncRNAs regulated by the top ten TFs in other independent cohorts. (A) The OS curves for GSE26193. (B) The OS curves for GSE9891. (C) The OS curves for GSE63885.

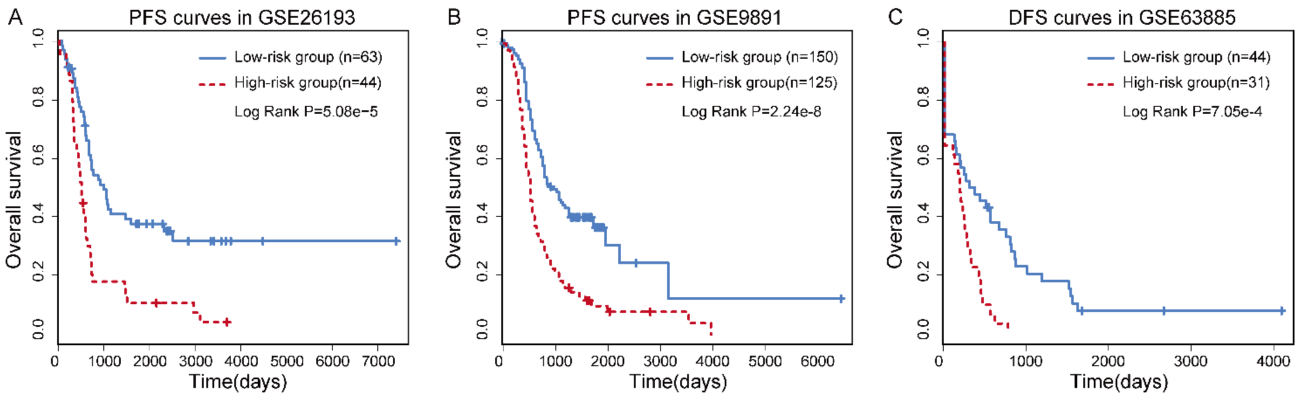

Figure S3. Using the same cut-off point identified from TCGA dataset to divide GEO datasets. (A) The PFS curves for GSE26193. (B) The PFS curves for GSE9891. (C) The DFS curves for GSE63885.

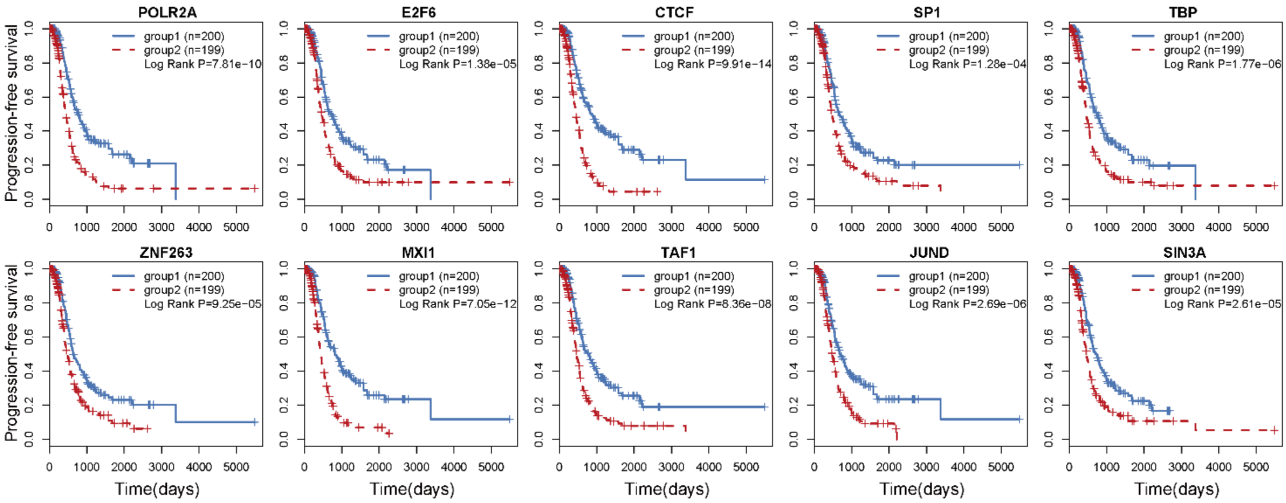

Figure S4. PFS analysis for lncRNAs regulated by the top ten TFs in TCGA dataset.

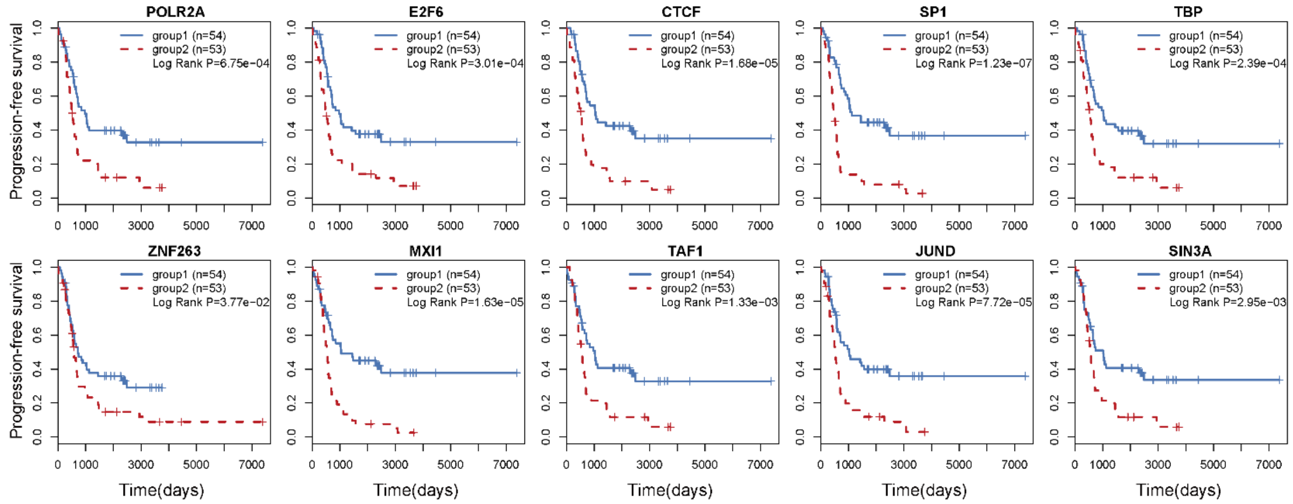

Figure S5. PFS analysis for lncRNAs regulated by the top ten TFs in GSE26193 dataset.

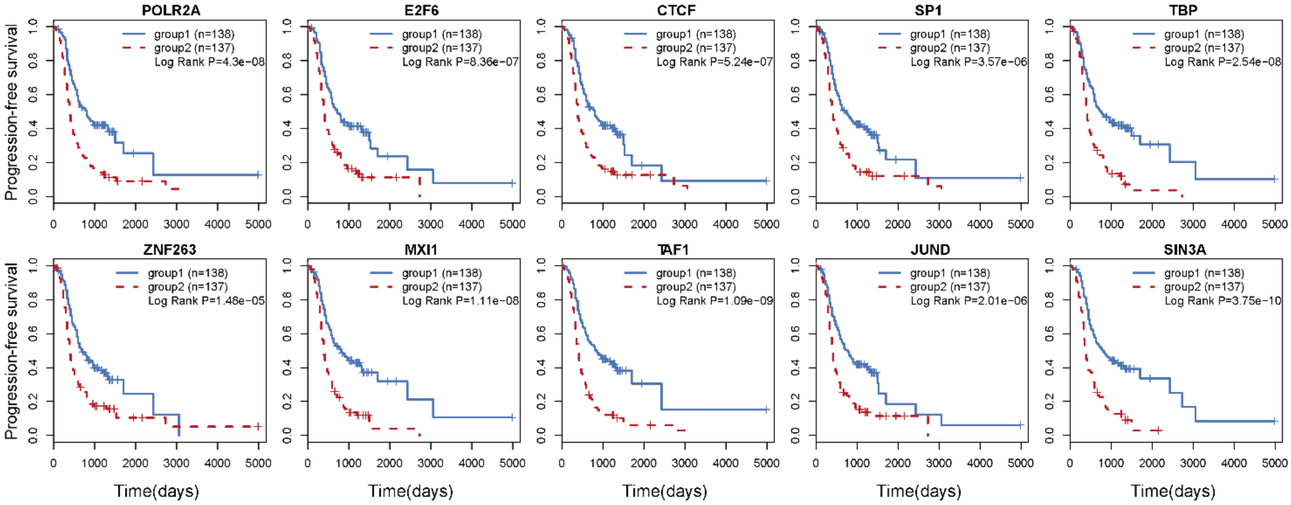

Figure S6. PFS analysis for lncRNAs regulated by the top ten TFs in GSE9891dataset.

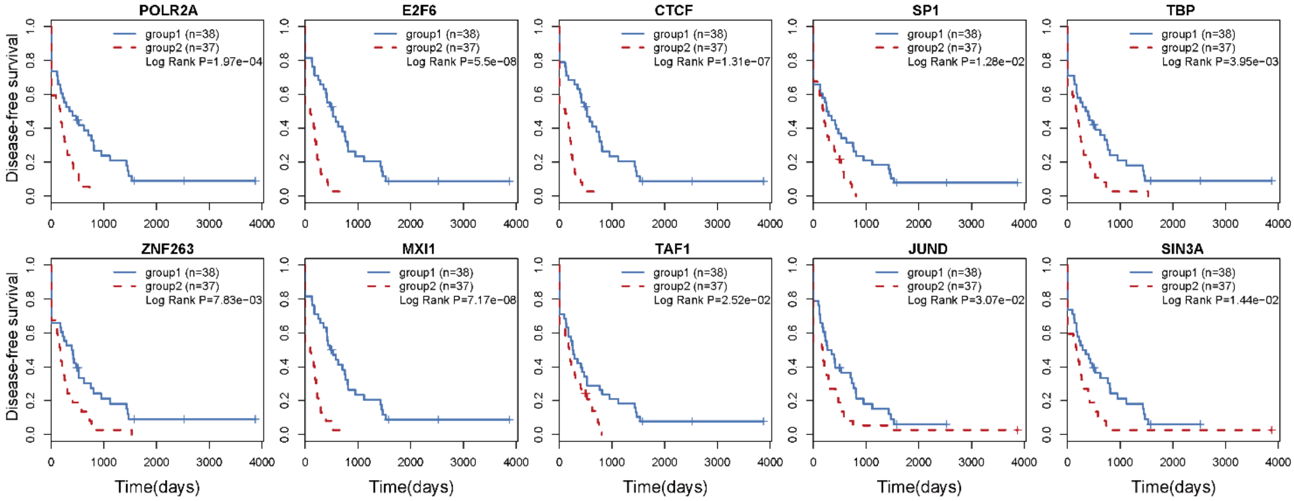

Figure S7. DFS analysis for lncRNAs regulated by the top ten TFs in GSE63885.

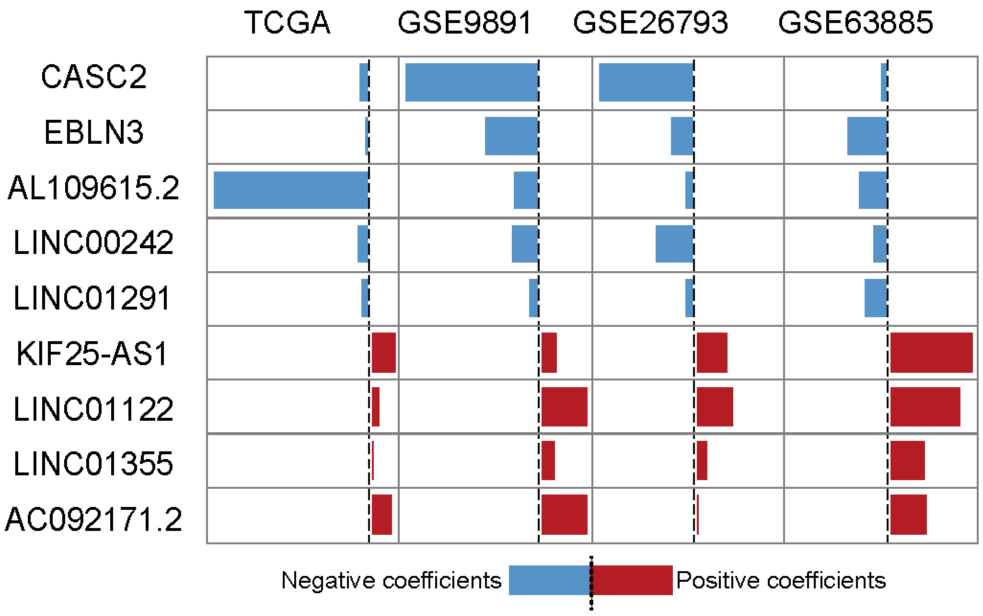

Figure S8. Nine lncRNAs have the same risk direction (positive or negative of regression coefficients) across 4 OV datasets.
