# Supplementary material for: Identification of potential prognostic TF‐associated lncRNAs for predicting survival in ovarian cancer
Source: J Cell Mol Med. 2018 Dec 13;23(3):1840–51. doi: 10.1111/jcmm.14084 (PMC6378234; doi:10.1111/jcmm.14084)
Supplement: Supplementary file 2 [file JCMM-23-1840-s002.pdf]

Table S1. qRT-PCR primers used in our study.

Primer	Sequence (5′–3′)
KIF25-AS1 forward	CCAGACTGAATGTGGGCGTAA
KIF25-AS1 reverse	ACACTCAAGGCTCAGATGACCAA
LINC01355 forward	TGGTCAGAATGCTCTAACTCTTATG
LINC01355 reverse	CAGAACTAGGTCACTACTTTGGGAA
AC092171.2 forward	CAAAAGCCTTGCGGAGTAGA
AC092171.2 reverse	ACAATTACGGCGTCTCGGA

Table S2. Univariable Cox regression analysis of the top ten TFs and overall survival in each dataset.

Datasets	TFs	HR (95%CI)	Coefficient	P-values
	POLR2A	1.675(1.501-1.870)	0.52	0
	E2F6	1.372(1.273-1.479)	0.32	1.11E-16
	CTCF	1.439(1.319-1.569)	0.36	2.22E-16
	SP1	2.266(1.843-2.786)	0.82	8.55E-15
TCGA	TBP	1.558(1.387-1.75)	0.44	7.51E-14
(n=399)	ZNF263	2.434(1.92-3.087)	0.89	2.06E-13
	MXI1	2.518(1.967-3.223)	0.92	2.23E-13
	TAF1	1.844(1.556-2.184)	0.61	1.52E-12
	JUND	2.427(1.886-3.123)	0.89	5.41E-12
	SIN3A	2.139(1.716-2.667)	0.76	1.41E-11
	POLR2A	1.774(1.463-2.152)	0.57	5.73E-09
	E2F6	1.708(1.392-2.097)	0.54	3.05E-07
	CTCF	1.523(1.293-1.793)	0.42	4.58E-07
	SP1	1.95(1.586-2.399)	0.67	2.51E-10
GSE9891	TBP	1.987(1.562-2.528)	0.69	2.24E-08
(n=278)	ZNF263	2.487(1.664-3.715)	0.91	8.75E-06
	MXI1	2.195(1.773-2.717)	0.79	5.21E-13
	TAF1	1.923(1.552-2.381)	0.65	2.08E-09
	JUND	2.382(1.638-3.463)	0.87	5.47E-06
	SIN3A	2.148(1.701-2.713)	0.76	1.41E-10
	POLR2A	1.475(1.262-1.724)	0.39	1.08E-06
	E2F6	1.252(1.123-1.396)	0.23	5.18E-05
	CTCF	1.385(1.221-1.572)	0.33	4.11E-07
	SP1	1.709(1.307-2.235)	0.54	9.14E-05
GSE26193	TBP	1.355(1.15-1.596)	0.30	2.78E-04
(n=107)	ZNF263	2.261(1.365-3.745)	0.82	1.53E-03
	MXI1	1.974(1.426-2.733)	0.68	4.14E-05
	TAF1	1.701(1.34-2.16)	0.53	1.28E-05
	JUND	1.924(1.497-2.472)	0.65	3.23E-07
	SIN3A	1.919(1.397-2.637)	0.65	5.82E-05
	POLR2A	1.287(1.156-1.432)	0.25	4.02E-06
	E2F6	1.368(1.202-1.557)	0.31	1.96E-06
	CTCF	1.498(1.296-1.731)	0.40	4.41E-08
	SP1	1.371(1.195-1.572)	0.32	6.32E-06
GSE63885	TBP	1.453(1.227-1.721)	0.37	1.47E-05
(n=75)	ZNF263	3.551(1.912-6.594)	1.27	5.99E-05
	MXI1	1.559(1.295-1.875)	0.44	2.59E-06
	TAF1	1.334(1.15-1.548)	0.29	1.44E-04
	JUND	1.512(1.163-1.967)	0.41	2.04E-03
	SIN3A	1.301(1.132-1.494)	0.26	2.07E-04

Table S3. Univariable Cox regression analysis of the top ten TFs and PFS/DFS in each dataset.

Datasets	TFs	HR (95%CI)	Coefficient	P-values
	POLR2A	1.631(1.447-1.839)	0.49	1.11E-15
	E2F6	1.613(1.383-1.881)	0.48	1.15E-09
	CTCF	1.776(1.579-1.998)	0.57	0.00E+00
	SP1	1.483(1.303-1.687)	0.39	2.27E-09
TCGA	TBP	1.932(1.585-2.354)	0.66	6.56E-11
(PFS, n=399)	ZNF263	1.877(1.5-2.35)	0.63	3.74E-08
	MXI1	2.516(2.026-3.123)	0.92	1.11E-16
	TAF1	1.727(1.468-2.031)	0.55	4.48E-11
	JUND	2.408(1.83-3.17)	0.88	3.61E-10
	SIN3A	1.544(1.319-1.807)	0.43	6.19E-08
	POLR2A	1.839(1.540-2.195)	0.61	1.56E-11
	E2F6	1.848(1.524-2.242)	0.61	4.46E-10
	CTCF	1.622(1.391-1.892)	0.48	7.19E-10
	SP1	2.066(1.672-2.553)	0.73	1.84E-11
GSE9891	TBP	2.055(1.669-2.529)	0.72	1.10E-11
(PFS, n=275)	ZNF263	2.474(1.767-3.466)	0.91	1.37E-07
	MXI1	2.053(1.697-2.483)	0.72	1.34E-13
	TAF1	1.961(1.603-2.398)	0.67	5.65E-11
	JUND	2.337(1.685-3.243)	0.85	3.76E-07
	SIN3A	2.155(1.726-2.69)	0.77	1.15E-11
	POLR2A	1.541(1.313-1.81)	0.43	1.29E-07
	E2F6	1.392(1.208-1.603)	0.33	4.66E-06
	CTCF	1.423(1.251-1.618)	0.35	7.90E-08
	SP1	1.816(1.418-2.326)	0.60	2.34E-06
GSE26193	TBP	1.44(1.188-1.746)	0.36	2.06E-04
(PFS, n=107)	ZNF263	2.172(1.286-3.668)	0.78	3.73E-03
	MXI1	1.714(1.333-2.205)	0.54	2.71E-05
	TAF1	1.593(1.291-1.965)	0.47	1.38E-05
	JUND	2.362(1.772-3.148)	0.86	4.48E-09
	SIN3A	1.699(1.305-2.212)	0.53	8.27E-05
	POLR2A	1.478(1.239-1.765)	0.39	1.50E-05
	E2F6	1.556(1.289-1.877)	0.44	3.99E-06
	CTCF	1.512(1.283-1.781)	0.41	7.67E-07
	SP1	1.498(1.217-1.843)	0.40	1.34E-04
GSE63885	TBP	1.757(1.325-2.331)	0.56	9.12E-05
(DFS, n=75)	ZNF263	2.191(1.229-3.905)	0.78	7.83E-03
	MXI1	2.048(1.5-2.796)	0.72	6.36E-06
	TAF1	1.435(1.166-1.765)	0.36	6.31E-04
	JUND	1.869(1.325-2.638)	0.63	3.69E-04
	SIN3A	1.561(1.216-2.004)	0.45	4.82E-04

Table S4. Significantly enriched biological processes and pathways for POLR2A-associating lncRNAs.

Groups	Enriched biological pathways and terms	P-values
KEGG Pathway	Phenylalanine, tyrosine and tryptophan biosynthesis (hsa00400)	1.22E-02
	Sulfur metabolism (hsa00920)	2.42E-02
	Axon guidance (hsa04360)	3.88E-02
	Selenocompound metabolism (hsa00450)	4.09E-02
	Phenylalanine metabolism (hsa00360)	4.09E-02
	2-Oxocarboxylic acid metabolism (hsa01210)	4.09E-02
GO	Negative regulation of integrin-mediated signaling pathway (GO:2001045)	4.92E-04
	Positive regulation of cell adhesion (GO:0045785)	1.62E-03
	Positive regulation of cell-substrate adhesion (GO:0010811)	2.79E-03
	Positive regulation of cell-cell adhesion (GO:0022409)	3.02E-03
	Positive regulation of cell adhesion mediated by integrin (GO:0033630)	3.74E-03
	Cell chemotaxis to vascular endothelial growth factor (GO:0090667)	4.96E-03
	Retrograde vesicle-mediated transport, Golgi to ER (GO:0006890)	8.42E-03

Table S5. LncRNAs regulated by TF PLOR2A.

Ensembl ID	Symbol
ENSG00000178977	LINC00324
ENSG00000177640	CASC2
ENSG00000233137	EBLN3
ENSG00000231881	AL109615.2
ENSG00000229214	LINC00242
ENSG00000204792	LINC01291
ENSG00000226137	BAIAP2-AS1
ENSG00000245768	AC092378.1
ENSG00000234492	RPL34-AS1
ENSG00000261839	AL358933.1
ENSG00000248975	AL133372.2
ENSG00000260588	AC027702.1
ENSG00000251323	AP003086.1
ENSG00000224812	TMEM72-AS1
ENSG00000233237	LINC00472
ENSG00000261423	TMEM202-AS1
ENSG00000258701	LINC00638
ENSG00000230590	FTX
ENSG00000246777	AC044802.1
ENSG00000226756	AC007365.1
ENSG00000260464	AL049796.1
ENSG00000261519	AC010542.4
ENSG00000182057	OGFRP1
ENSG00000228223	HCG11
ENSG00000188660	LINC00319
ENSG00000250312	ZNF718
ENSG00000185168	LINC00482
ENSG00000260805	AC092803.2
ENSG00000260655	CTA-250D10.23
ENSG00000251161	AC020661.1
ENSG00000260633	AC010207.1
ENSG00000261504	LINC01686
ENSG00000233760	AC004947.1
ENSG00000237658	RP5-968D22.3
ENSG00000223823	LINC01342
ENSG00000205181	LINC00654
ENSG00000178947	SMIM10L2A
ENSG00000259863	SH3RF3-AS1
ENSG00000260913	LINC01254
ENSG00000260597	AC012531.1
ENSG00000188185	LINC00265
ENSG00000260261	AC124944.3
ENSG00000237021	RP3-486I3.7
ENSG00000261334	AL353803.4
ENSG00000248265	AC023794.1
ENSG00000180422	LINC00304
ENSG00000237438	CECR7
ENSG00000223573	TINCR
ENSG00000175873	AC004840.9
ENSG00000229921	KIF25-AS1
ENSG00000233723	LINC01122
ENSG00000261326	LINC01355
ENSG00000230733	AC092171.2
